# A high-content morphological screen identifies novel microRNAs that regulate neuroblastoma cell differentiation

**DOI:** 10.18632/oncotarget.1703

**Published:** 2014-02-28

**Authors:** Zhenze Zhao, Xiuye Ma, Tzu-Hung Hsiao, Gregory Lin, Adam Kosti, Xiaojie Yu, Uthra Suresh, Yidong Chen, Gail E. Tomlinson, Alexander Pertsemlidis, Liqin Du

**Affiliations:** ^1^ Greehey Children's Cancer Research Institute, UT Health Science Center at San Antonio, TX; ^2^ Graduate School of Biomedical Sciences, UT Health Science Center at San Antonio, TX; ^3^ Department of Epidemiology and Biostatistics, UT Health Science Center at San Antonio, TX; ^4^ Department of Pediatrics, UT Health Science Center at San Antonio, TX; ^5^ Department of Cellular and Structural Biology, UT Health Science Center at San Antonio, TX

**Keywords:** neuroblastoma, microRNA, high-content screen, differentiation, differentiation therapy

## Abstract

Neuroblastoma, the most common extracranial solid tumor of childhood, arises from neural crest cell precursors that fail to differentiate. Inducing cell differentiation is an important therapeutic strategy for neuroblastoma. We developed a direct functional high-content screen to identify differentiation-inducing microRNAs, in order to develop microRNA-based differentiation therapy for neuroblastoma. We discovered novel microRNAs, and more strikingly, three microRNA seed families that induce neuroblastoma cell differentiation. In addition, we showed that microRNA seed families were overrepresented in the identified group of fourteen differentiation-inducing microRNAs, suggesting that microRNA seed families are functionally more important in neuroblastoma differentiation than microRNAs with unique sequences. We further investigated the differentiation-inducing function of the microRNA-506-3p/microRNA-124-3p seed family, which was the most potent inducer of differentiation. We showed that the differentiation-inducing function of microRNA-506-3p/microRNA-124-3p is mediated, at least partially, by down-regulating expression of their targets *CDK4* and *STAT3*. We further showed that expression of miR-506-3p, but not miR-124-3p, is dramatically upregulated in differentiated neuroblastoma cells, suggesting the important role of endogenous miR-506-3p in differentiation and tumorigenesis. Overall, our functional screen on microRNAs provided the first comprehensive analysis on the involvements of microRNA species in neuroblastoma cell differentiation and identified novel differentiation-inducing microRNAs. Further investigations are certainly warranted to fully characterize the function of the identified microRNAs in order to eventually benefit neuroblastoma therapy.

## INTRODUCTION

Neuroblastoma is the most common solid tumor of infancy and the most common extracranial solid tumor of childhood, accounting for more than 7% of childhood cancers and 15% of cancer-related childhood deaths [1, 2]. Neuroblastoma arises from the neural crest cell precursors of the sympathetic nervous system that fail to differentiate [2, 3] – this provides the basis for differentiation therapy, an approach to induce malignant cells to differentiate into mature cells, thereby leading to cell growth arrest and apoptosis [2, 4-6]. However, only a limited number of differentiation agents have been successfully used to treat neuroblastoma. The differentiation agent 13-cis-retinoic acid (RA) is currently the standard of care for post-remission maintenance therapy in high-risk neuroblastoma [2]. Although such treatment has resulted in a significant increase in patient survival, more than 50% of the treated patients still develop recurrence [7, 8]. Such poor outcomes demand the development of new differentiation agents. Unfortunately, the mechanisms that result in the loss of differentiation ability of neuroblastoma cells are poorly understood, which poses an obstacle to such development. Therefore, identifying additional differentiation agents largely relies on the discovery of new targetable biological molecules that play critical roles in neuroblastoma differentiation.

High-throughput screening approaches significantly facilitate the discovery of novel anti-cancer drugs and drug targets. More recently, high-content screens (HCSs) based on automated cell imaging have been developed. However, current HCSs generally either use genetic engineered cell lines expressing fluorescent signals or involve staining of fixed cells [9, 10], which are generally time-consuming and consequently limit their broad applications to drug discoveries. We aim to develop a non-fluorescence, live-cell based HCS approach for identifying neuroblastoma differentiation-inducing agents. Neurite outgrowth, which is easily detectable under the microscope, is a well-recognized morphological differentiation marker of neuroblastoma cells *in vitro* [11-14]. While undifferentiated cells usually show no visible neurites, fully differentiated neuroblastoma cells form neurites that are four to five times the length of the cell body. This differentiation trait facilitates the design of a functional HCS assay to directly identify substances that induce neuroblastoma cell differentiation. In this study, we developed such an approach and applied it in a screen for microRNAs (miRNAs) that induce differentiation.

miRNAs are endogenously expressed small RNAs that play a critical role in tumorigenesis [15-19]. The therapeutic potential of either exogenously increasing cellular miRNAs levels with synthetic miRNA mimics, or inactivating endogenous miRNAs with synthetic miRNA inhibitors has been demonstrated in previous studies [20-22]. The role of miRNAs in neuroblastoma differentiation and tumorigenesis has been implicated[23-31], which suggests the potential of developing novel miRNA-targeting approaches to neuroblastoma differentiation therapy[32], and warrants a comprehensive understanding of the involvement of miRNAs in neuroblastoma cell differentiation. However, there has been no concerted effort to comprehensively investigate the functions of the miRNA species in neuroblastoma differentiation. By applying the HCS that we developed, we investigated the recently identified human miRNAs and identified differentiation-inducing miRNAs that have not been discovered previously.

## RESULTS

### A HCS approach for measuring neuroblastoma cell differentiation is developed based on neurite quantification

**Figure 1:**
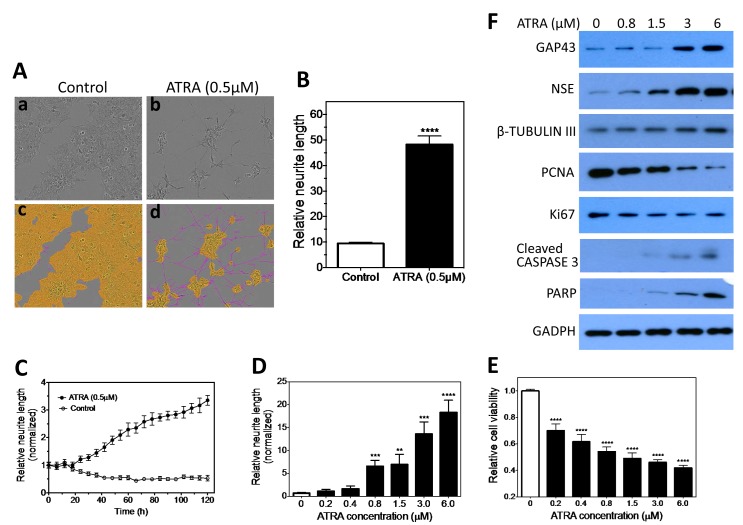
Neurite length is a quantifiable differentiation marker of BE(2)-C cells 2,500 cells were plated in 96-well plates and cultured overnight. Cells were then treated with ATRA or carrier (DMSO, control) and placed into the IncuCyte for detecting neurite outgrowth. 9 images were taken from each well to allow for statistical analysis. Relative neurite length is defined as neurite length per cell body area. A, ATRA induces neurite outgrowth. Shown are representative phase-contrast images for cells treated with (a) carrier or (b) ATRA for 5 days, and (c, d) the same images analyzed to define neurites (pink) and cell body areas (yellow). B, Quantification shows that ATRA significantly increases the relative neurite length compared to control. C, Relative neurite lengths increase in a time-dependent manner during ATRA-induced cell differentiation. Neurite lengths were normalized to the starting time point (0 h). D, Dose-dependent effect of ATRA on neurite outgrowth. Shown are the results after treating with ATRA for 5 days. E, Dose-dependent effect of ATRA on cell viability. Cells were treated with different concentrations of ATRA, and cell viability was determined after 5 days. F, Dose-dependent effect of ATRA on the protein expression levels of cell differentiation markers GAP43, NSE and β-TUBULIN III, cell proliferation markers Ki67 and PCNA, and apoptotic markers cleaved CASPASE 3 and PARP, with GAPDH protein levels used as a loading control. Cells were treated with ATRA as above, and protein levels were determined by Western blots after 5 days. **, *p*<0.01; ***, *p*<0.001; ****, *p*<0.0001.

Neurite outgrowth is well recognized as a morphological hallmark of neuroblastoma cell differentiation *in vitro* [11-14]. This facilitates the development of a HCS approach to identify differentiation-inducing agents based on quantification of neurite outgrowth. Neuroblastoma cell line BE(2)-C shows easily detectable neurite outgrowth upon induced differentiation by all-trans retinoic acid (ATRA). As shown in Figure 1A, ATRA (b) induces dramatic neurite outgrowth in BE(2)-C compared to control (a), and the neurites and cell body area can be clearly defined (c, d). Quantification (Figure 1B) shows that ATRA significantly increases the relative neurite length compared to control. In addition, ATRA induces neurite elongation in both time- and dose-dependent manners (Figure 1C-D and Suppl. Figure 1). Correspondingly, ATRA decreases cell viability (Figure 1E), stimulates expression of neuroblastoma differentiation markers (i.e., growth associated protein 43 (GAP43), neuron specific enolase (NSE) and β-TUBULIN III) [33-35], inhibits expression of cell proliferation markers (i.e., PCNA and Ki67), and increases expression of apoptosis markers (i.e., cleaved CASPASE 3 and PARP) (Figure 1F) in dose-dependent manners. These results indicate that neurite length is a reliable quantitative marker of BE(2)-C cell differentiation, and therefore can be used to compare the efficacy of differentiation-inducing agents. This was the basis of our HCS protocol (Suppl. Figure 2) for identifying novel differentiation-inducing miRNAs.

### HCS identifies novel miRNAs that induce neuroblastoma cell differentiation

**Figure 2:**
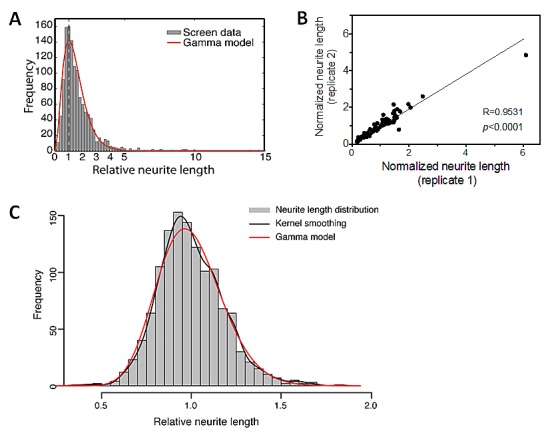
HCS screening of miRNA mimics identifies miRNAs that induce neurite outgrowth in BE(2)-C cells A-B, 2,500 cells were reverse-transfected with 25 nM miRNA mimics in 96-well plates. After 4 days of transfection, relative neurite lengths were quantified as above. A, The distribution of post-normalized neurite length measurements (grey histogram) for individual miRNA mimics from the screen and the fitted Gamma distribution curve (red line, α=3.27, β=0.444) generated by the method described in Suppl. Figure 3. B, Correlation between two independent neurite length measurements for one library plate. Cells were transfected with 25 nM mimics in two independent 96-well plates. Normalized neurite lengths were measured as above. Correlation of neurite length between the two plates was analyzed using two-tailed Pearson Correlation with *p*<0.05 considered significant. C, The distribution of the neurite lengths for untreated BE(2)-C cells. 2,500 Cells were plated into each well in 96 well plates. After 4 days, neurite lengths were analyzed as above. Shown are the neurite length distribution histogram (grey), the empirical density curve (black line), and the fitted Gamma distribution curve (red line, α=30.23, β=0.033). Using the *P*<0.05 threshold, Kolmogorov-Smirnov goodness-of-fit test for Gamma model validity did not reject the null hypothesis (*p*=0.16), which support that the neurite length distribution fits Gamma model.

Using our HCS protocol, we screened a library of miRNA mimics (Dharmacon) in BE(2)-C cells. Figure 2A shows the neurite length distribution (grey histogram) associated with individual miRNA mimics. Replicate screens for one library plate from two independent transfections show that the results are highly reproducible (Figure 2B) (R=0.95, *p*<0.0001), supporting the reliability of the screen. As shown in Figure 2A, the neurite length distribution following treatment with miRNA mimics is asymmetric; a small number of miRNAs are identified as dramatically increasing neurite lengths on the far right side of the distribution. In order to examine the neurite length distribution of unaffected cells, 13 plates of untreated BE(2)-C cells were measured (Figure 2C). Kolmogorov-Smirnov goodness-of-fit test for Gamma model validity indicates that the neurite length distribution fit Gamma model (*p*=0.16), which informs us to use this model (red line in Figure 2A) to assess the effect of individual miRNAs, as illustrated in Suppl. Figure 3. Fourteen miRNA mimics were identified as significantly increasing neurite length using False Discovery Rate (FDR) threshold <0.01 (Table 1). Using the same threshold, 0 hits were generated from untreated cells, indicating the specificity of the analysis approach. Among the 14 miRNAs, several were related to neuroblastoma cell differentiation in previous studies (Table 1) [27, 28, 36, 37], demonstrating the sensitivity of our HCS approach.

**Table 1:** Fourteen miRNA mimics identified from HCS as inducing neurite outgrowth using a FDR threshold <0.01 Shown are the (a) miRNA name, (b) fold change of neurite length relative to unaffected cells, (c) *p* value, (d) FDR, (e) mature sequences of the miRNAs with seed sequences underlined and seed family grouping indicated in parentheses, and (f) references that have reported the involvements of the corresponding miRNAs in differentiation.

(a) miRNA	(b) Neurite length (Mean±SD)	(c) p value	(d) FDR	(e) Mature sequence 5'-3'	(f) Ref
hsa-miR-124-3p	14.55±4.75	1.11E-11	1.37E-08	UAAGGCACGCGGUGAAUGCC (1)	27, 37
hsa-miR-135b-5p	14.02±4.29	3.26E-11	2.02E-08	UAUGGCUUUUCAUUCCUAUGUGA	
hsa-miR-506-3p	10.04±3.41	1.05E-07	4.34E-05	UAAGGCACCCUUCUGAGUAGA (1)	
hsa-miR-34a-5p	9.49±3.18	3.11E-07	8.81E-05	UGGCAGUGUCUUAGCUGGUUGU (2)	
hsa-miR-103a-3p	9.43±2.67	3.56E-07	8.81E-05	AGCAGCAUUGUACAGGGCUAUGA (3)	28
hsa-miR-450b-3p	9.26±2.64	4.94E-07	1.02E-04	UUGGGAUCAUUUUGCAUCCAUA	
hsa-miR-449a	9.16±2.47	6.03E-07	1.07E-04	UGGCAGUGUAUUGUUAGCUGGU (2)	
hsa-miR-2110	9.00±2.40	8.21E-07	1.27E-04	UUGGGGAAACGGCCGCUGAGUG	
hsa-miR-34b-5p	8.41±2.16	2.66E-06	3.66E-04	UAGGCAGUGUCAUUAGCUGAUUG	
hsa-miR-107	7.60±1.98	1.27E-05	1.55 E-03	AGCAGCAUUGUACAGGGCUAUCA (3)	36
hsa-miR-3714	7.56±1.91	1.37E-05	1.55E-03	GAAGGCAGCAGUGCUCCCCUGU	
hsa-miR-449b-5p	7.40±1.85	1.89E-05	1.95E-03	AGGCAGUGUAUUGUUAGCUGGC (2)	
hsa-miR-137	7.03±1.36	3.83E-05	3.65E-03	UUAUUGCUUAAGAAUACGCGUAG	19, 34
hsa-miR-3937	6.93±1.05	4.61E-05	4.08E-03	ACAGGCGGCUGUAGCAAUGGGGG	

**Figure 3:**
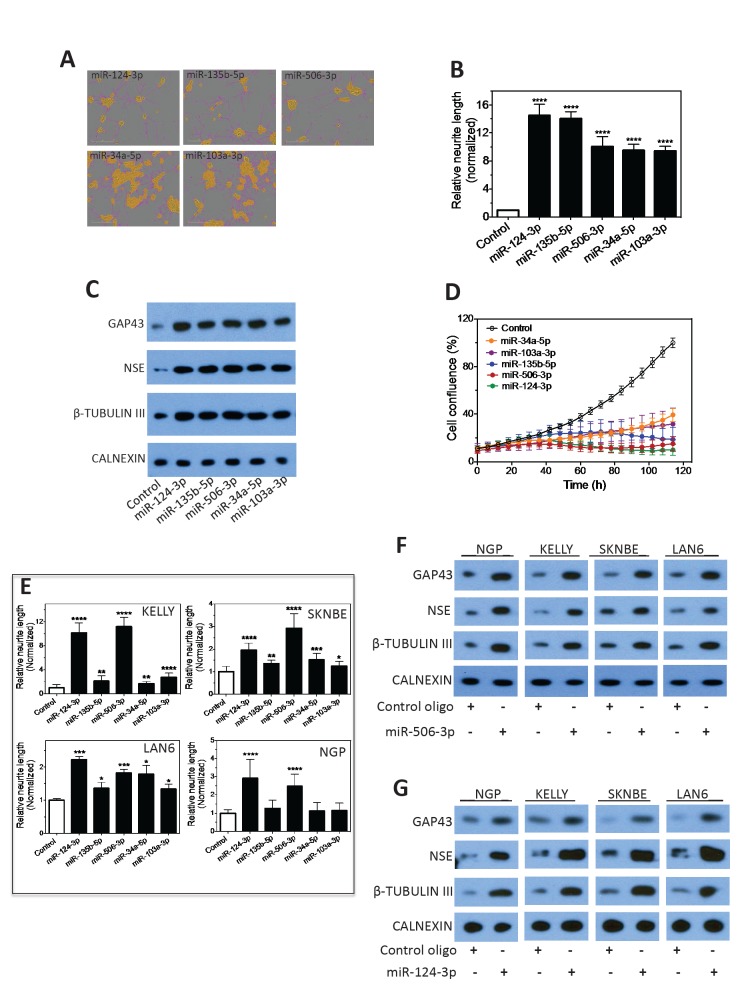
Characterization of the effect of the top 5 neurite-inducing miRNA mimics on cell differentiation and growth in multiple neuroblastoma cell lines A-B, Effects of the identified top 5 miRNA mimics on neurite outgrowth. BE(2)-C cells were transfected with 25 nM miRNA mimics or control for 4 days, and relative neurite lengths were quantified as above. Shown are representative cell images analyzed to define neurite and cell body areas (A) and neurite length quantifications (B). C, Effects of the 5 miRNA mimics on expression of differentiation markers, with CALNEXIN protein levels used as a loading control. BE(2)-C cells were transfected with 25 nM of the indicated miRNA mimics or control, and proteins levels were examined after 4 days. D, Effects of the 5 miRNA mimics on cell proliferation rate. E, Effect of the 5 miRNAs on neurite outgrowth in multiple cell lines. Cells were transfected with 25 nM miRNA mimics or control, and neurite lengths were quantified as above after 5 days. F-G, Effects of miR-506-3p (F) and miR-124-3p (G) mimics on expression of differentiation markers in multiple cell lines. *, *p*<0.05; **, *p*<0.01; ***, *p*<0.001; ****, *p*<0.0001.

**Figure 4:**
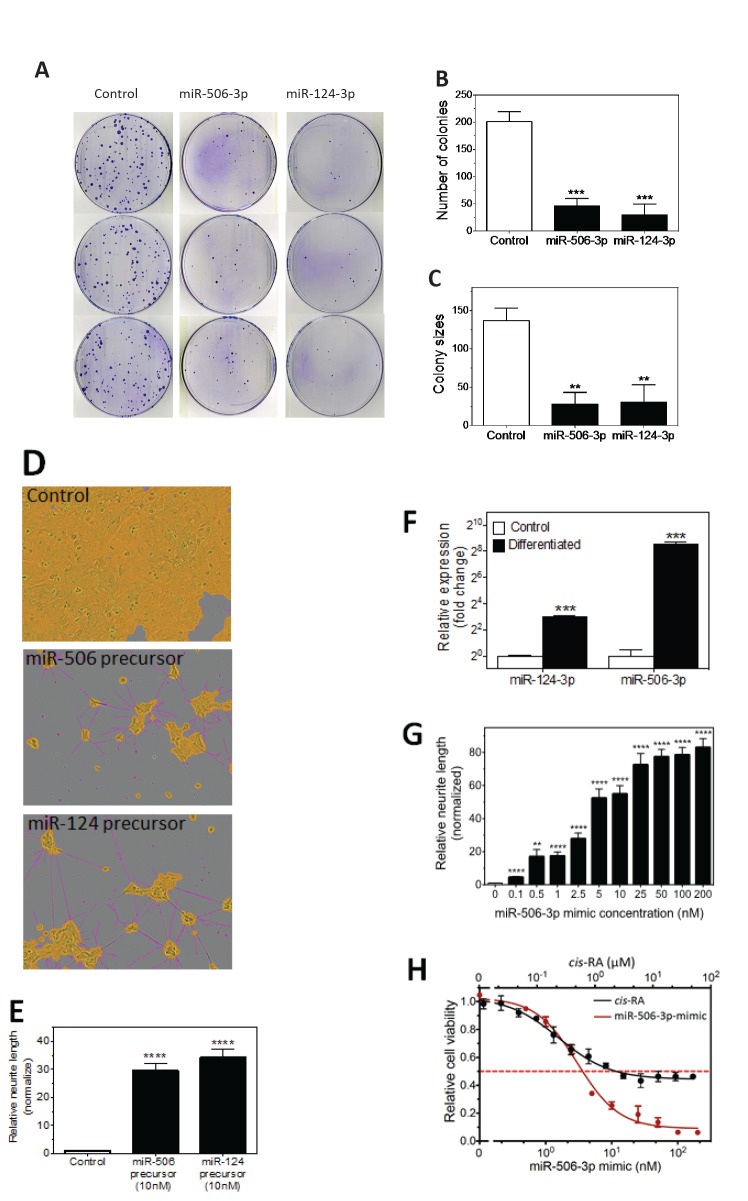
Characterization of the tumor suppressive function of miR-506-3p/miR-124-3p family A-C, Colony formation assay as a function of miR-124-3p and miR-506-3p mimics. BE(2)-C cells were transfected with the 25 nM of the indicated oligos and colony formation was examined as above. Shown are (A) plate images of colony formation and quantified colony numbers (B) and sizes (C). D-E, Effect of miR-124-3p and miR-506-3p precursors on neurite outgrowth. BE(2)-C cells were transfected with or without 10 nM miRNA precursors. Shown are (D) Representative images analyzed to define neurites and cell body areas after 4 days transfections and (E) quantification of relative neurite lengths. F, Effect of cell differentiation on endogenous expression of miR-506-3p and miR-124-3p in BE(2)-C cells. G, Dose-dependent effect of miR-506-3p mimic on neurite outgrowth in BE(2)-C cells. Cells were transfected with different concentrations of miR-506-3p mimic, and relative neurite lengths were quantified as above after 4 days. Neurite lengths were normalized to control (0 nM). H, Dose-dependent effect of miR-506-3p mimic and *cis-*RA on cell viability in BE(2)-C cells. Cells were transfected with different concentrations of miR-506-3p mimic for 4 days or treated with different concentrations of *cis-*RA for 5 days, and cell viability was determined as above. *, *p*<0.05; **, *p*<0.01; ***, *p*<0.001; ****, *p*<0.0001.

We next focused on characterizing the differentiation-inducing function of the top 5 microRNAs that are most potent in inducing neurite outgrowth (Figure 3A-B). We showed that, comparing to control, the 5 miRNAs induce expression of differentiation markers and dramatically decrease BE(2)-C cell growth rate (Figure 3C-D), demonstrating that true cell differentiation and growth arrest are induced. We further investigated the 5 miRNAs in additional neuroblastoma cell lines with different genetic backgrounds (Suppl. Table 1). Among the 5 miRNAs, miR-506-3p and miR-124-3p, which belong to the same seed family (defined as a group of miRNAs that share common seed sequence, Table 1), have the most potent effect on neurite growth (Figure 3E and Suppl. Figure 4). Correspondingly, the two miRNAs dramatically induce expression of differentiation markers in all the tested cell lines (Figure 3F-G). We further show that miR-506-3p and miR-124-3p mimics significantly reduce the ability of BE(2)-C to form colonies, indicating their long-term capacity to inhibit cell proliferation (Figure 4A-C). To exclude the possibility that the induced cell differentiation and growth arrest are caused by off-target effects of the specific chemical designs of miRNA mimics, we examined the effect of miR-506-3p and miR-124-3p precursors (Ambion) on differentiation. The miRNA precursors are partially double-stranded RNAs designed to mimic the functions of the endogenous miRNAs, in contrast to the fully complementary double-stranded design of miRNA mimics. Figure 4D-E shows that the precursors significantly induced neurite outgrowth, recapitulating the results with miRNA mimics. This indicates that the differentiation-inducing function of miR-506-3p and miR-124-3p mimics is unlikely caused by off-target effect. Overall, the above results demonstrate the general and potent effect of miR-506-3p/miR-124-3p on inducing differentiation, suggesting the potential of restoring miR-506-3p and miR-124-3p expressions as a novel differentiation therapeutic strategy to treat neuroblastoma.

To further examine the potential pathophysiological relevance of endogenous miR-506-3p and miR-124-3p in regulating neuroblastoma differentiation, we measured their expression levels in differentiated BE(2)-C cells. Figure 4F shows that expressions of both miRNAs are significantly increased in differentiated cells. However, the overexpression of miR-506-3p (380.1±48.4 fold) is much more dramatic than that of miR-124-3p (8.1±0.4 fold). These results indicate that endogenous miR-506-3p expression in neuroblastoma cells is highly regulated and suggest that, relative to miR-124-3p, endogenous miR-506-3p is likely to be a more dominant driving force in controlling cell differentiation. We further observe that miR-506-3p mimic induces neurite outgrowth and decreases cell viability in a dose-dependent manner, and that the induction of neurite outgrowth is significant at a concentration as low as 0.1 nM (Figure 4G-H, Suppl. Figure 5). Figure 4H also shows that the cytotoxic effect of miR-506-3p mimic is much more potent (reducing cell viability to 6.3±0.3% when reaching plateau) than 13-cis retinoic acid (cis*-*RA) (42.6±3.7%), a differentiation-agent currently used to treat neuroblastoma patients [7]. Altogether, these results demonstrate the potent function of miR-506-3p in inducing differentiation and in reducing cell survival and growth. The role of miR-506-3p in neuroblastoma tumorigenesis has not been investigated previously; we are the first to discover its differentiation inducing function in neuroblastoma.

From the screen, we also identified miRNAs that significantly reduce cell survival but do not induce differentiation (Suppl. Figure 6). This indicates that miRNAs modulate neuroblastoma cell survival and growth through distinct mechanisms, and cell differentiation is not a prerequisite for cell death or growth arrest, which supports the functional specificity of the identified differentiation-inducing miRNAs in regulating differentiation.

### miRNA seed families that are potent inducers of cell differentiation are discovered from HCS

**Figure 5:**
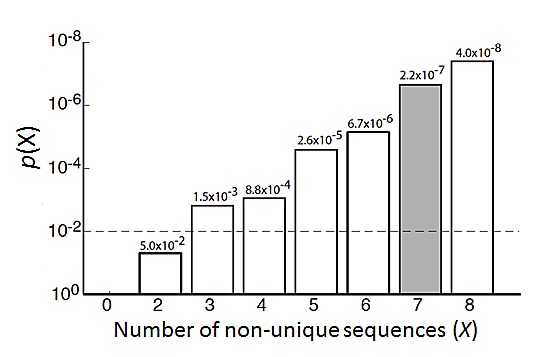
Enrichment analysis of seed families in a set of 14 miRNAs by random permutation Grey bar shows the probability (*p*=2.2×10^−7^) that at least 7 non-unique seed sequences appear in a randomly selected set of 14 miRNAs.

miRNA seed sequences are central in determining their target genes [38-41]. We therefore first analyzed the seed sequences of the identified differentiation-inducing miRNAs. Surprisingly, as shown in Table 1, three miRNA seed families, which accounts for 7 miRNAs, are identified within the 14 miRNAs. Enrichment analysis by random permutation shows that the probability that ≥7 non-unique seed sequences appear in a set of 14 randomly drawn miRNAs from the miRNA mimic library is *p*=2.2×10^−7^ (Grey bar in Figure 5), indicating that miRNA seed-sequences families are significantly overrepresented in the identified 14 miRNAs. Further investigation of the remaining 7 miRNAs shows that the seed sequences of miR-135b-5p (shares seed sequence with miR-135a-5p), miR-34b-5p (shares with miR-449c-5p and miR-2682-5p) and miR-450b-3p (shares with miR-769-3p) are not unique. In addition, seed sequence family 2 includes another miRNA, miR-34c-5p, which is not identified in the top 14 candidates. Close examination of the screen results indicates that miR-34c-5p, miR-135a-5p and miR-449c-5p also increase the neurite lengths (Suppl. Figure 7), ranking as 17^th^, 43^th^ and 128^th^ in the screen (miR-2682-5p was not in the library), although the effect of miR-449c-5p did not reach statistical significance using *p*< 0.05 threshold. miR-769-3p does not induce neurite outgrowth, and, however, is the only exception among the identified differentiation-inducing seed families.

### The identified differentiation-inducing miRNAs are predicted to target distinct spectra of genes involved in neuroblastoma differentiation

**Figure 6:**
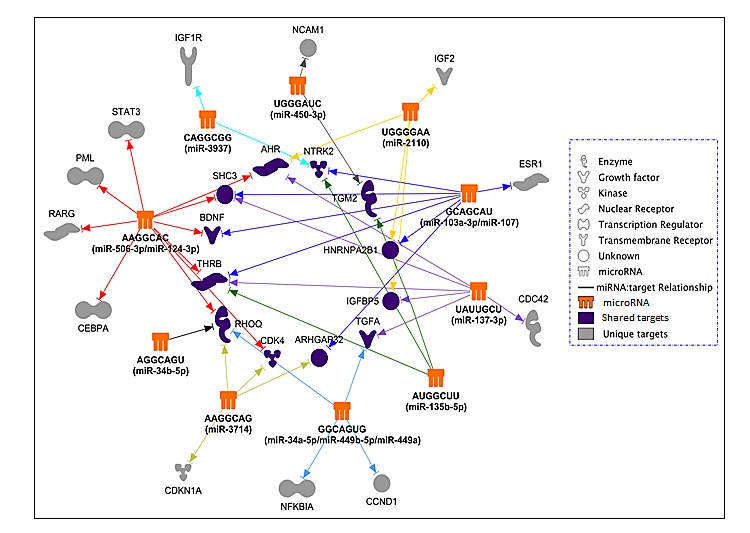
The predicted differentiation-inducing targetome network for the identified 14 differentiation-inducing miRNAs The 14 miRNAs were grouped into 10 seed-sequence groups. Predicted targets previously reported as involved in neuroblastoma differentiation were used to create the targetome network.

The above observations lead us to identify potential miRNA targets based on seed sequence matches. We first identified 48 genes that have been previously demonstrated to regulate neuroblastoma differentiation (Suppl. Table 2.) using Ingenuity Pathway Analysis (IPA) (Ingenuity System). Not surprisingly, further IPA miRNA target analysis indicates that each of the miRNAs/seed families is predicted to target multiple genes involved in neuroblastoma differentiation (Figure 6), among which the miR-506-3p/miR-124-3p family is predicted to target 10 of the 48 genes. The results also show that, although the predicted targets of these miRNAs/seed-sequence families overlap, each miRNA/seed-sequence family has a unique targetome, suggesting that they are likely to induce cell differentiation through distinct, but overlapping pathways.

### CDK4 and STAT3 play a role in mediating the differentiation-inducing function of miR-506-3p/miR-124-3p family

**Figure 7:**
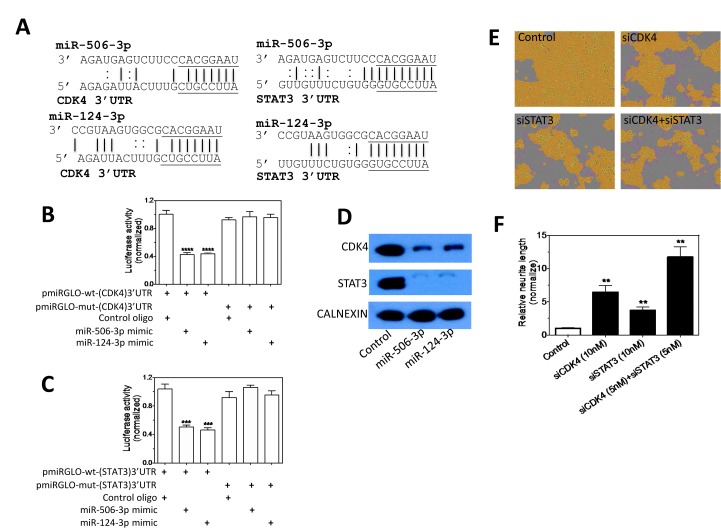
Validation of CDK4 and STAT3 as direct targets that mediate the differentiation-inducing function of miR-506-3p and miR-124-3p A, The predicted interactions between miR-506-3p/miR-124-3p and the target sites in the 3'UTR of STAT3 and CDK4 mRNAs. The seed sequences are underlined. B-C, Validation of the target sites of miR-506-3p and miR-124-3p in the 3'UTRs of (B) CDK4 and (C) STAT3 by luciferase reporter assay. BE(2)-C cells were co-transfected with the indicated vectors and miRNA mimics or control oligo. After 72 h of transfection, cells were lysed and luciferase activity was measured. Shown are normalized luciferase activities of different treatment groups. D, miR-506-3p and miR-124-3p overexpression down-regulate endogenous CDK4 and STAT3 protein expression levels. Cells were transfected as above for 4 days, and protein levels were measured by Western blots. E-F, Effect of CDK4 and STAT3 knockdown on neurite outgrowth in BE(2)-C cells. Cells were transfected with the indicated oligos, and neurite lengths were measured as above after 4 days transfection. Shown are representative cell images analyzed to define neurites and cell body areas (G) and quantification of neurite length under the indicated treatment conditions (H). **, *p*<0.01; ***, *p*<0.001; ****, *p*<0.0001.

We further investigated the targets of miR-506-3p/miR-124-3p family. Table 2 shows that the expression changes of the 10 predicted target genes of miR-506-3p/miR-124-3p induced by their overexpressions are almost identical (Table 2), further demonstrating that the seed-sequence is dominant in determining the miRNA function. The two miRNAs dramatically down-regulate two of the ten predicted targets, *CDK4* and *STAT3*. Figure 7A shows the interactions of the two miRNAs with the predicted target sites in the 3'UTR of CDK4 and STAT3. We validated their target sites in the 3'UTRs of CDK4 and STAT3 using luciferase reporter assays (Figure 7B-C). We further show that overexpressions of the two miRNAs down-regulate endogenous CDK4 and STAT3 protein levels (Figure 7D). Figure 7E-F shows that individual repression of *CDK4* and *STAT3* expression induces neurite outgrowth, and that their combined repression has an enhanced effect on neurite outgrowth relative to individual repression. These results indicate that CDK4 and STAT3 mediate, at least partially, the differentiation-inducing function of miR-506-3p/miR-124-3p, and suggest that the effect of miR-506-3p/miR-124-3p on cell differentiation are most likely mediated by concordantly down-regulating multiple target genes.

**Table 2:** Changes of expression for the 10 predicted targets of the miR-506-3p/miR-124-3p family induced by miR-506-3p and miR-124-3p overexpression Shown are the gene name and the fold change of mRNA expression levels (miRNA mimic *vs.* control) induced after 24 h transfection with (1) miR-506-3p mimic and (2) miR-124-3p mimic. *, Shown are average expressions detected by multiple probes in the microarray.

Gene	Gene expression*	
	(1) miR-506-3p	(2) miR-124-3p
CDK4	0.39	0.43
STAT3	0.75	0.75
CEBPA	0.88	0.86
PML	1.01	1.00
RARG	1.02	1.03
BDNF	1.04	1.06
THRB	1.04	1.06
RHOQ	1.07	1.13
SHC3	1.08	1.07
AHR	1.19	1.17

## DISCUSSION

In this study, we developed a HCS approach to facilitate the discovery of novel differentiation-inducing agents for neuroblastoma. Several HCS approaches based on quantification of neurite outgrowth have been described [33, 42-44]. However, quantifications of neurite outgrowth in these approaches were either based on cell lines engineered to express fluorescent reporters or involve staining of fixed cells [33, 42-44]. These approaches are generally time-consuming. In addition, whether the extent of neurite outgrowth in neuroblastoma cells is a reliable marker to evaluate the potency of differentiation-inducing agents was not clearly characterized. Here we showed that, in neuroblastoma cell line BE(2)-C, neurite length of differentiated cells is quantifiable based on phase-contrast images of live cells and is a reliable marker of the extent of cell differentiation. This supports us to develop a HCS approach to examine neuroblastoma cell differentiation by quantifying neurite outgrowth, and we for the first time show that the neurite length distribution of untreated cells fit the Gamma model – this informs us to use this model to perform statistical analysis on neurite-based HCS. Our screen of a library of miRNA mimics demonstrates that our screening and statistical analysis methods are specific and sensitive for identifying differentiation-inducing agents. We expect that this approach will facilitate future discovery of novel differentiation-inducing drugs and drug targets for treating neurobloastoma.

Our study provides the first comprehensive and direct functional analysis of miRNA species in inducing neuroblastoma cell differentiation. Our screen not only recapitulated several previous findings, but also identified miRNAs that have not been known to regulate neuroblastoma differentiation. For example, we are the first to identify the function of miR-506-3p in promoting neuroblastoma cell differentiation. Our further investigations show that endogenous miR-506-3p expression was dramatically increased in differentiated neuroblastoma cells. In addition, published data have indicated the tissue-specific expression of miR-506 in adrenal gland, the primary tissue of origin for neuroblastoma [45]. This evidence altogether implicated the important role of miR-506-3p in neuroblastoma pathogenesis. Overall, our comprehensive analysis of miRNAs in neuroblastoma cell differentiation is the first step towards elucidating the entire picture of miRNA involvement in differentiation. We expect that further investigation on the identified individual miRNAs will provide a better understanding of the mechanisms of neuroblastoma tumorigenesis.

In our HCS, several miRNAs that were previously reported to induce neuroblastoma cell differentiation were not identified as potent inducers of cell differentiation [29, 30]. One of the possible explanations is the cell context-specific variation of the function of these miRNAs, in that our screen was conducted in a different cell line from those used in previous studies. Further studies are certainly warranted to compare the differentiation-inducing function of these previously reported miRNAs with the miRNAs identified in our study in a larger panel of neuroblastoma cell lines and in *in vivo* studies, and to compare their clinical relevance in neuroblastoma tumorigenesis in clinical studies.

Another intriguing finding in our study is the overrepresentation of a set of miRNA seed families in our identified differentiation-inducing miRNAs. This observation is beyond providing convincing evidence supporting the reliability of our HCS approach. It leads us to an interesting speculation: the conservation of seed sequence among different miRNAs located at different genomic regions may be an important evolution trait that is selected by nature to guard the normal cell differentiation process during development; if one miRNA fails its expression in cells, expression of another seed-family member will perform similar function to prevent differentiation error. If this is true, we expect that miRNA seed sequence families are more likely to be involved in cell differentiation than miRNAs with unique sequences. There are certainly other possible explanations. Whether miRNA seed families are also overrepresented in other differentiation/development processes and what are the pathophysiological significances of this overrepresentation are certainly interesting questions to pursue in the future.

Previous studies have demonstrated the promise of synthetic miRNA mimics as therapeutic agents in cancer treatment [20, 22]. Our identification of novel differentiation-inducing miRNA mimics therefore directly provides a group of novel candidates potentially applicable to treat neuroblastoma. Our identification of several seed-sequence families that are potent differentiation inducers reinforces the notion that miRNA seed sequences play the key role in defining their biological function, which provides the rationale for developing novel synthetic seed sequence-based oligos as differentiation-inducing agents. By replacing the nucleic acids in the non-seed positions while keeping the seed sequence unchanged, various seed sequence-based synthetic oligos can be designed and tested *in vitro* and *in vivo*, in order to identify the optimal design that has the most potent effect on neuroblastoma differentiation and has minimal non-specific cytotoxicity on normal cells and tissues.

Currently, differentiation agents are limited to be used for post-remission maintenance therapy in high-risk neuroblastoma [2]. One of the reasons for this limitation is that the current available differentiation agents are not as potent as other anti-cancer agents in ablating cancer cells. However, our study show that miR-506-3p mimic reduced neuroblastoma cell viability to a much greater extent than cis*-*RA, raising the possibility of applying differentiation-inducing miRNA mimics to remission induction therapy. Further investigations in *in vivo* models are certainly warranted to examine the therapeutic efficacy of miR-506-3p mimics.

In this study, we preliminarily analyzed the predicted targetomes of the identified top 14 differentiation-inducing miRNAs using informatics tools, and took a small step toward validating the predicted targets of miR-506-3p/miR-124-3p family. Our investigation suggests that the differentiation-inducing function of a miRNA is likely mediated by concordantly down-regulating multiple targets. Our current analysis is limited to genes that are known to regulate neuroblastoma differentiation. It is highly likely that there are undiscovered targets of these miRNAs that play important roles in mediating their differentiation-inducing functions. Indeed, our expression array analyses indicate that miR-506-3p/miR-124-3p overexpression down-regulates many more of their predicted targets than we investigated in this study (data not shown). Whether these targets play roles in mediating their differentiation-inducing function warrants further investigation.

In summary, we have established a HCS platform to screen for novel differentiation-inducing substances in neuroblastoma cells. We have identified novel miRNAs and miRNA seed families that induce neuroblastoma cell differentiation. Our study not only provides the first comprehensive understanding of the role of miRNAs in neuroblastoma differentiation, but also provides novel leads for developing miRNA-based differentiation agents for neuroblastoma treatment. We expect that further investigation based on our findings will not only promote better understanding of the mechanisms of neuroblastoma tumorigenesis, but may also provide new therapeutic strategies for treating neuroblastoma.

## MATERIALS AND METHODS

### Materials

ATRA and cis-RA were from Sigma (St Luis, MO, USA). Dharmacon miRNA mimic library and individual miRNA mimics were from Thermo Fisher Scientific (Rockford, IL, USA). miRNA precursors were purchased from Ambion (Foster City, CA, USA). Rabbit anti-GAP43, anti-NSE, and anti-β-TUBULIN III were from Abcam (Cambridge, MA, USA). Rabbit anti-CALNEXIN, anti-GAPDH and goat anti-rabbit secondary antibody conjugated with horseradish peroxidase (HRP) were from Santa Cruz (Dallas, TX, USA). Rabbit anti-PARP (cleaved), anti-CASPASE-3, anti-STAT3, and anti-CDK4, were from Cell Signaling (Danvers, MA, USA). Rabbit anti-Ki67 was from Millipore (Billerica, MA, USA).

### Cell lines

BE(2)-C cells were purchased from the ATCC. Other cell lines were obtained from the cell line repository at the Greehey Children's Cancer Research Institute. Cells were grown in DMEM/F12 supplemented with 10% fetal bovine serum.

### Detection and quantification of neurite outgrowth

Cells were plated and treated in 96-well plates. For detecting neurite outgrowth, cells were placed into ZOOM IncuCyte Imaging System (Essen BioScience) and cell images were taken under 20X microscopic magnification. For detecting neurite outgrowth in a time-dependent manner, cell images were taken every 6 h. The neurite lengths associated with each treatment were calculated using the neurite definition defined for each specific cell line using the NeuroTrack system (Essen BioScience).

### Analysis of HCS data

The relative neurite length associated with cells in each well on the screen plates was determined as above. In order to allow for plate-to-plate comparison, neurite length associated with each well in each plate was first internally normalized to the mean of the corresponding plate, and multiple screen plates were then aggregated together to generate the neurite length distribution. The data was then further analyzed to determine the distribution of the unaffected cells as described below, and to identify differentiation-inducing miRNA mimics as illustrated in Suppl. Figure 3.

### Kolmogorov-Smirnov goodness-of-fit test for Gamma model validity

In order to examine whether the neurite length distribution of the untreated cells fit Gamma distribution, Gaussian kernel smoothing was first performed to generate the empirical density curve based on the neurite length distribution histogram of the untreated cells, and Gamma model parameters (α, β) were then estimated by minimizing the area in between empirical and Gamma distribution curves. Statistical significance of the fitness to Gamma distribution was examined by Kolmogorov-Smirnov goodness-of-fit test, with *p*<0.05 considered as rejecting the null hypothesis that the neurite length distribution fits Gamma distribution.

### Western blots

Cell lysates were prepared using RIPA buffer. Protein concentration was determined using the Pierce BCA assay (Thermo Fisher Scientific). For electrophoresis, equal amounts of cell lysate were resolved by SDS-PAGE and transferred to Immun-Blot PVDF membranes (Bio-Rad Laboratories). Membranes were blocked and probed with primary antibodies to specific proteins. Bound antibodies were detected with secondary antibodies conjugated with horseradish peroxidase (HRP) visualized by enhanced chemiluminescent (ECL) substrate (Pierce).

### Cell growth rate assay

Cells were plated in 96-well plates and were treated with specified conditions. Cells were placed into the IncuCyte imaging system and cell confluence was monitored every 6 h for 120 h. Cell growth curves were derived by comparing the cell confluences at different time points.

### Cell viability assay

Cells were plated in 96-well format and treated as specified. After culturing for 120 h, cell viability was determined using the CellTiter-Glo Luminescent Cell Viability Assay (Promega).

### Colony formation assay

Cells were transfected as specified and cultured overnight. 500 cells were re-plated in 10 cm dishes. After 14 days, colonies were visualized by staining with 1% crystal violet. Colony numbers and sizes were analyzed using Image J (NIH, Bethesda, MD).

### Enrichment analysis of miRNA seed families

We use random permutation to examine whether miRNA seed families are significantly enriched in the identified set of differentiation-inducing miRNAs. The miRNA mimic library includes mimics for 1231 human miRNAs listed in miRBase 16.0. Among these miRNAs, 900 miRNAs have unique seed sequences; 331 miRNAs share seed sequences with at least one other miRNA, constituting 125 seed sequence families. To calculate the probability of miRNAs from the same seed families randomly appears in a set of 14 miRNAs, 14 miRNAs were randomly drawn from the 1231 miRNAs, and the number of non-unique seed sequences within the 14 miRNAs is counted (X). 10^8^ permutations were run to determine the probability that at least × non-unique seed sequences appear in a randomly selected set of 14 miRNAs (*p*(*X*)).

### miRNA target prediction and pathway analysis

miRNA target sites in 3'UTRs were predicted based on seed sequence complementarity and were identified using the IPA Pathway analysis function, which identifies any 7-nucleotide region (3'-5') in a given 3'UTR completely complementary to the seed sequence of a miRNA (2^nd^ - 8^th^ or 1^st^ - 7^th^ nucleotides, 5'-3') as a potential target site of this miRNA. To identify targets that potentially mediate the differentiation-inducing function of our identified miRNAs, we first identified the genes that have been known to relate to neuroblastoma differentiation using IPA. From this gene list, we identified the predicted targets for each miRNA and generated the predicted miRNA:target interaction network mediating the differentiation-inducing function of the identified miRNAs.

### mRNA and miRNA expression

Total RNA was isolated as previously described [46]. mRNA expression profiling was done using the Illumina mRNA WG-6 v3 microarray platform. miRNA expression was measured by qRT-PCR using TaqMan microRNA Assays (Life Technologies) with average expression of RNU44 RNA, RNU19 RNA, GAPDH mRNA and 18s rRNA used as controls for normalizing RNA loading.

### Luciferase reporter assay

The segments of the wildtype 3'UTRs for *CDK4* and *STAT3* containing the predicted target sites of miR-506-3p and miR-124-3p were cloned from human genomic DNA. Mutant constructs were generated with the seed target site (GUGCCUU) replaced by CACGGUU. The 3'UTRs were cloned downstream of the firefly luciferase coding sequences into the pmirGLO dual-luciferase reporter (Promega), a vector containing both firefly and Renilla luciferase cDNAs under the control of separate promoter/terminator systems. The firefly luciferase was used as the primary reporter for miRNA regulation of the 3'UTR. The Renilla luciferase is an internal control for normalization. BE(2)-C cells were co-transfected with luciferase reporters (0.8 ng/ul) and miRNA mimics or control oligonucleotide (oligo) (25 nM). Luciferase activities were measured after 72 h using the Dual-Glo Luciferase Assay System (Promega). Firefly luciferase activity was normalized to Renilla luciferase activity to evaluate the effect of the miRNAs.

### Statistical analysis

For HCS, *p*-value for neurite length associated with each miRNA mimic was evaluated by assuming the distribution of neurite lengths follows a Gamma distribution, and FDR was determined by Benjamini-Hochberg correction method for multiple tests [47]. We consider a miRNA with FDR<0.01 as significantly inducing neurite outgrowth. For all other conditions, the statistical significance for each treatment was determined using *t*-test by comparing the treatment group with control, with *p*< 0.05 considered statistically significant.

## SUPPLEMENTARY FIGURES AND TABLES




