# Supplementary material for: A high-content morphological screen identifies novel microRNAs that regulate neuroblastoma cell differentiation
Source: Oncotarget. 2014 Feb 28;5(9):2499–512. doi: 10.18632/oncotarget.1703 (PMC4058022; doi:10.18632/oncotarget.1703)
Supplement: Supplementary file 1 [file oncotarget-05-2499-s001.pdf]

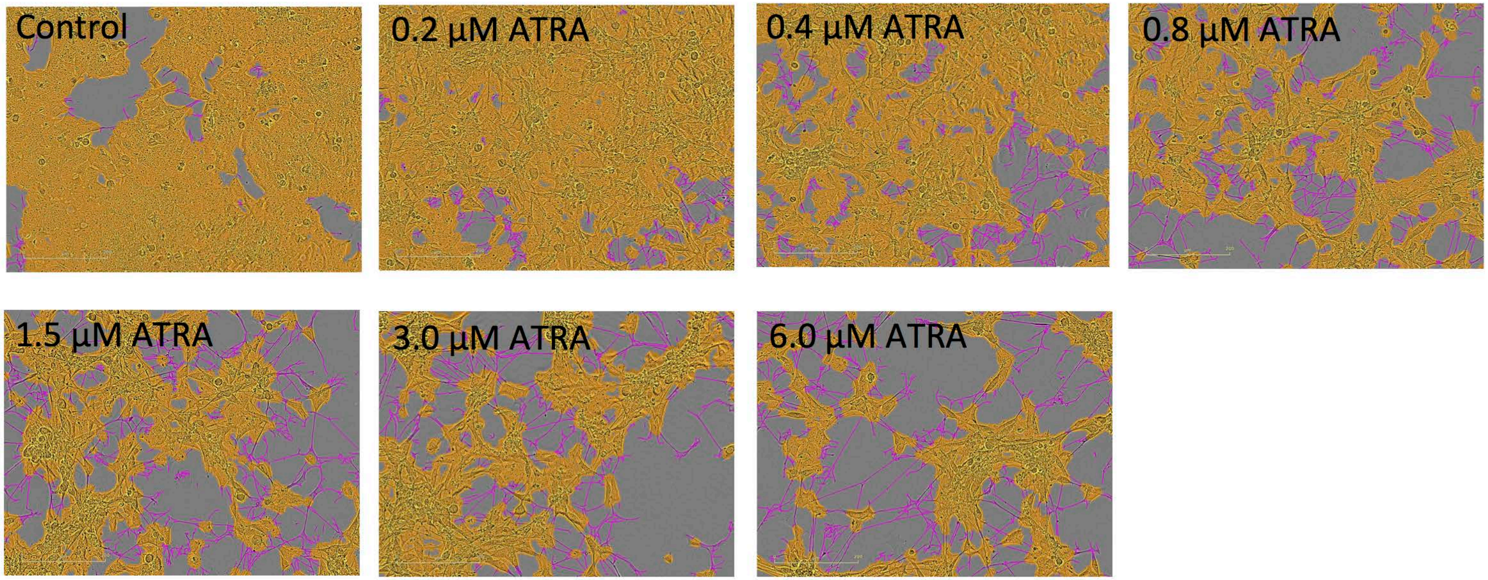

Suppl. Figure 1. Representative images showing the dose-dependent effect of ATRA on neurite outgrowth after 5 days of treatment. Shown are the images analyzed to define neurites and cell body areas.

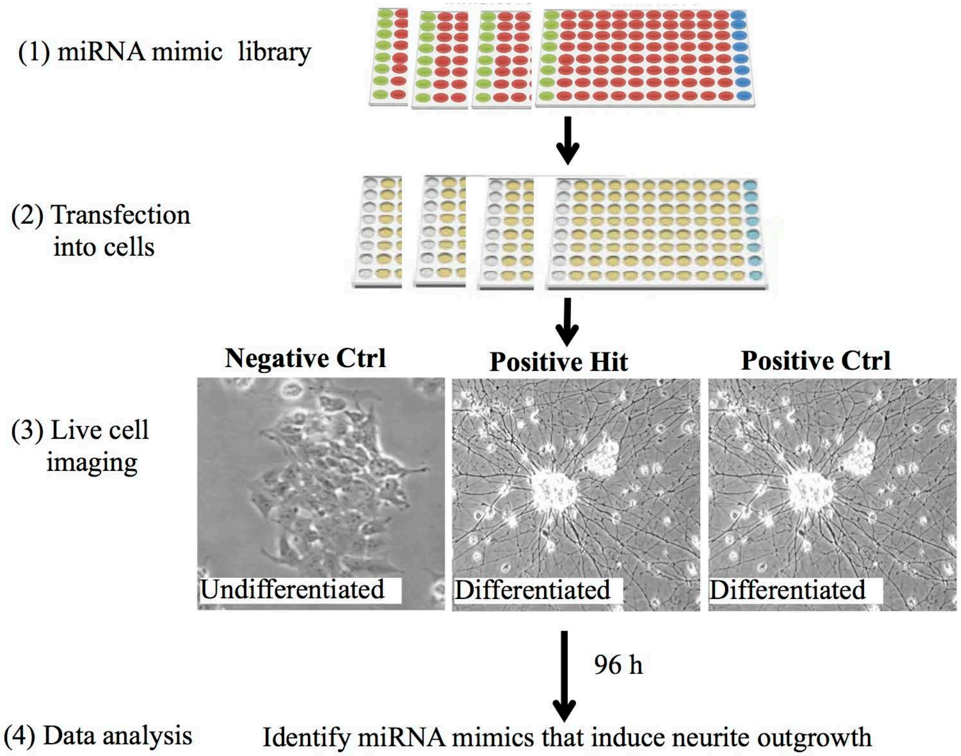

Suppl. Figure 2. Experimental design of the HCS. (1) In the library plates, columns 2-11 contain miRNA mimics, each of which raises the intracellular level of one human miRNA. Control treatments, including ATRA (0.5µM) as a positive control for cell differentiation, control mimic oligo (Dharmacon) as a negative control for cell differentiation, and siPLK1 (5nM) as a control of transfection efficiency were included in columns 1 and 12 of each library plate. (2) BE(2)-C cells were transfected with the mimic library at a final concentration of 25 nM. (3) Cells were then placed into the IncuCyte for measuring neurite outgrowth. (4) The neurite lengths associated with each miRNA mimic were analyzed after 96 h transfection for identifying differentiation-inducing miRNA mimics.

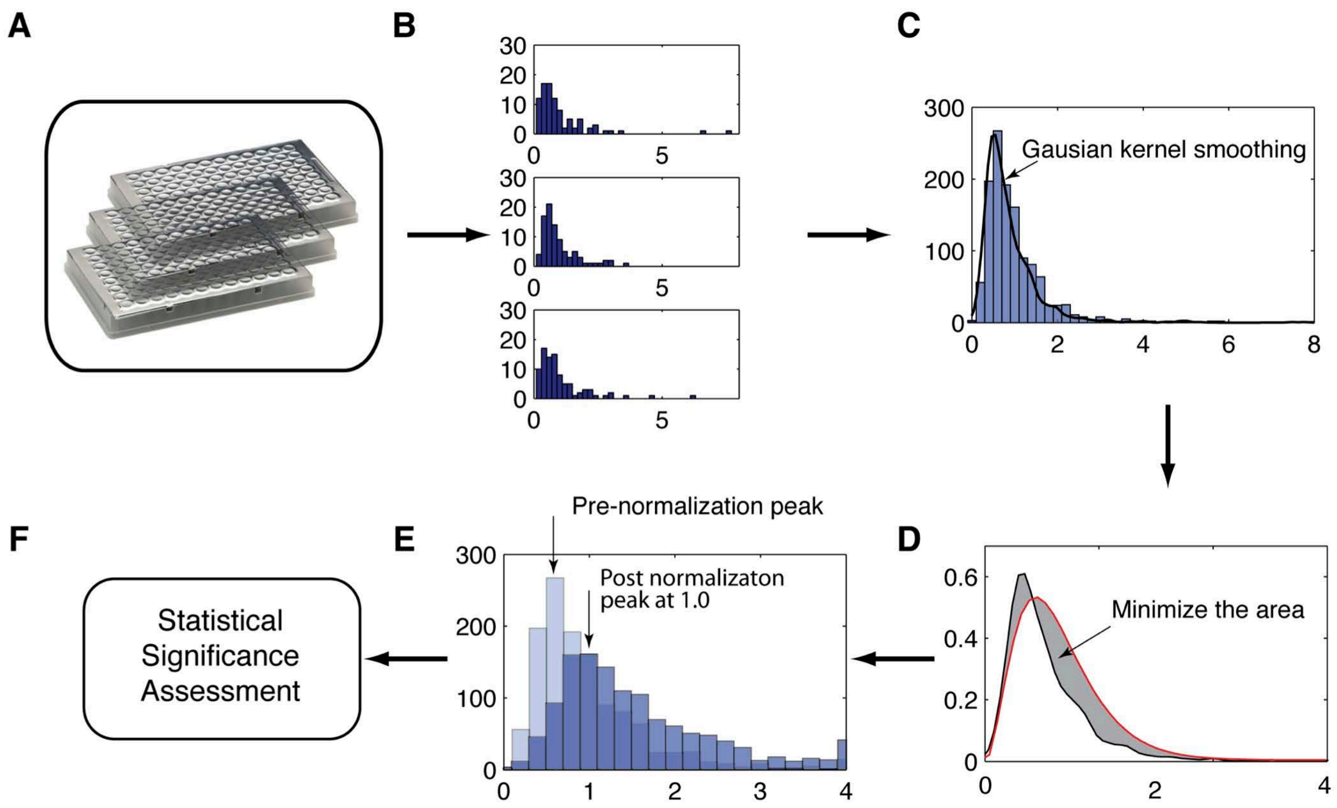

Suppl. Figure 3. The normalization approach for the miRNA mimic HCS data. A-B, Internal normalization of each library plate. The relative neurite length associated with each miRNA mimic in each library plate was internally normalized to the mean of the corresponding library plate. **C-D,** Estimation of Gamma distribution model parameters. The neurite length distribution of the whole library was generated by pooling all plates together (C, blue histogram). The empirical density curve (C-D, black line) was generated by Gaussian Kernel Smoothing and the fitted Gamma distribution curve (D, red line) were generated with the Gamma distribution parameters (a=3.27, b=0.270) estimated by minimizing the area in between empirical and Gamma distribution curves. The mode (the neurite length at the peak) of the Gamma model was (a -1)b=0.6129. **E,** Normalization of the library peak to 1.0. In order to quantify the effect of individual miRNAs on neurite outgrowth, the normalized neurite lengths from (**A**) were re-normalized by dividing the neurite length associated with each miRNA by the mode (0.6129) to effectively move the peak of the neurite length distribution to 1.0, assuming that the majority of the miRNAs surrounding the peak do not affect neurite lengths. Therefore, in the post-normalized dataset, the value associated with each miRNA mimic is the fold change of neurite length induced by this miRNA relative to unaffected cells. The parameters of Gamma distribution fitted to the post-normalized dataset are a=3.271, b=0.444. **F**, The statistical significance (p value) of the effect of each miRNA on neurite outgrowth was assessed by evaluating Gamma cumulative distribution to the right of the miRNA.

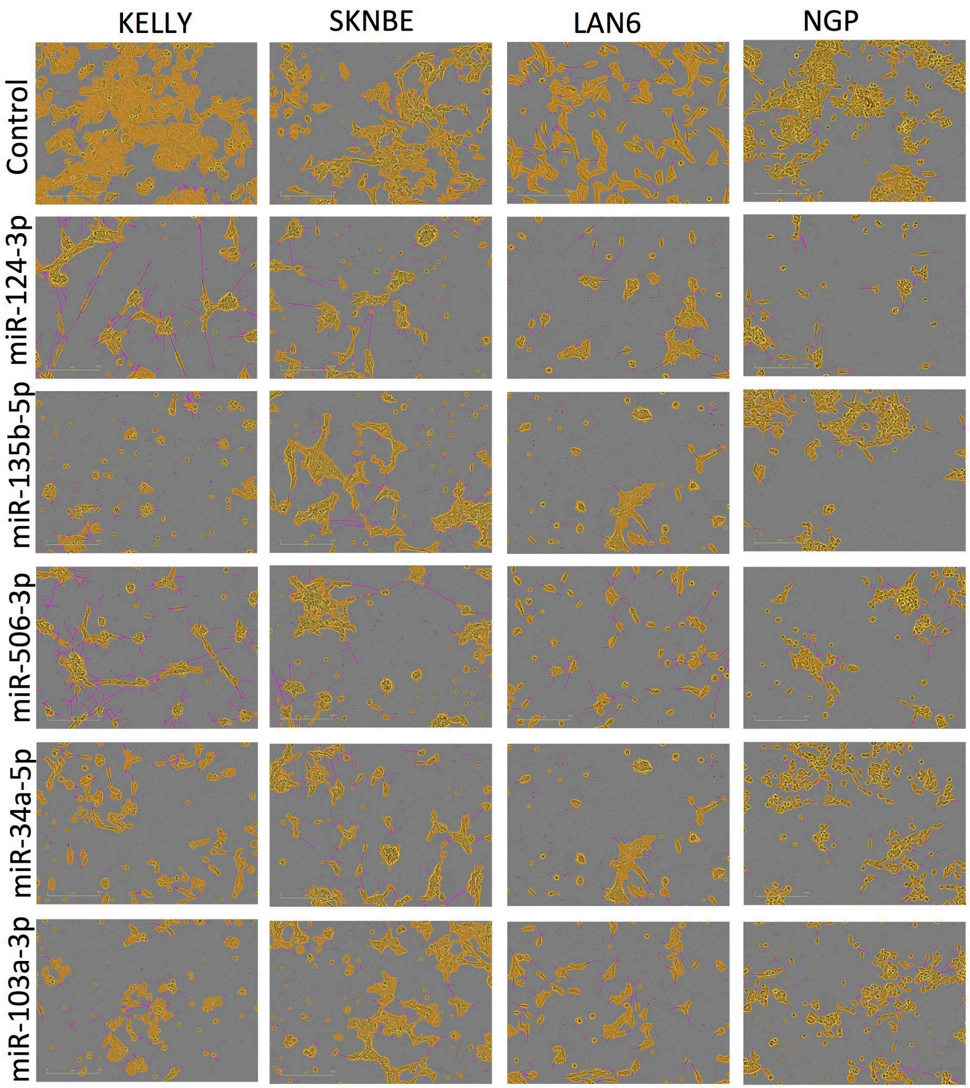

Suppl. Figure 4. Effect of the identified top 5 differentiation-inducing miRNAs mimics on differentiation in multiple neuroblastoma cell lines. Shown are the images analyzed to define neurites and cell body areas.

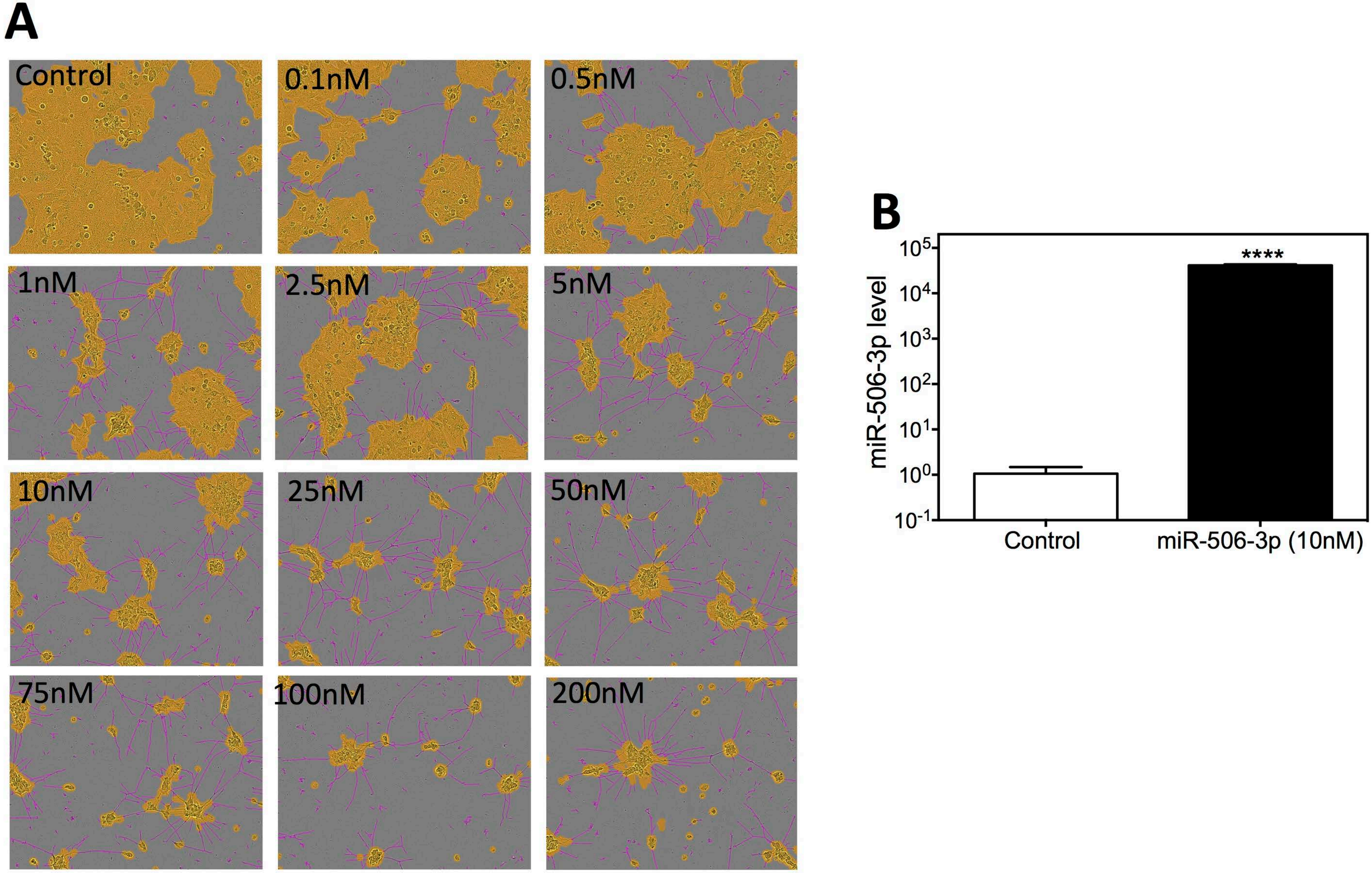

Suppl. Figure 5. Dose-dependent effect of miR-506-3p mimic on neurite outgrowth in BE(2)-C cells. A, Shown are representative images analyzed to define neurites and cell body areas at each of the indicated miR-506-3p mimic concentrations. **B,** Expression level of miR-506-3p after 24 h transfection with 10nM miR-506-3p mimic in BE(2)-C cells. ****, $p<0.0001$.

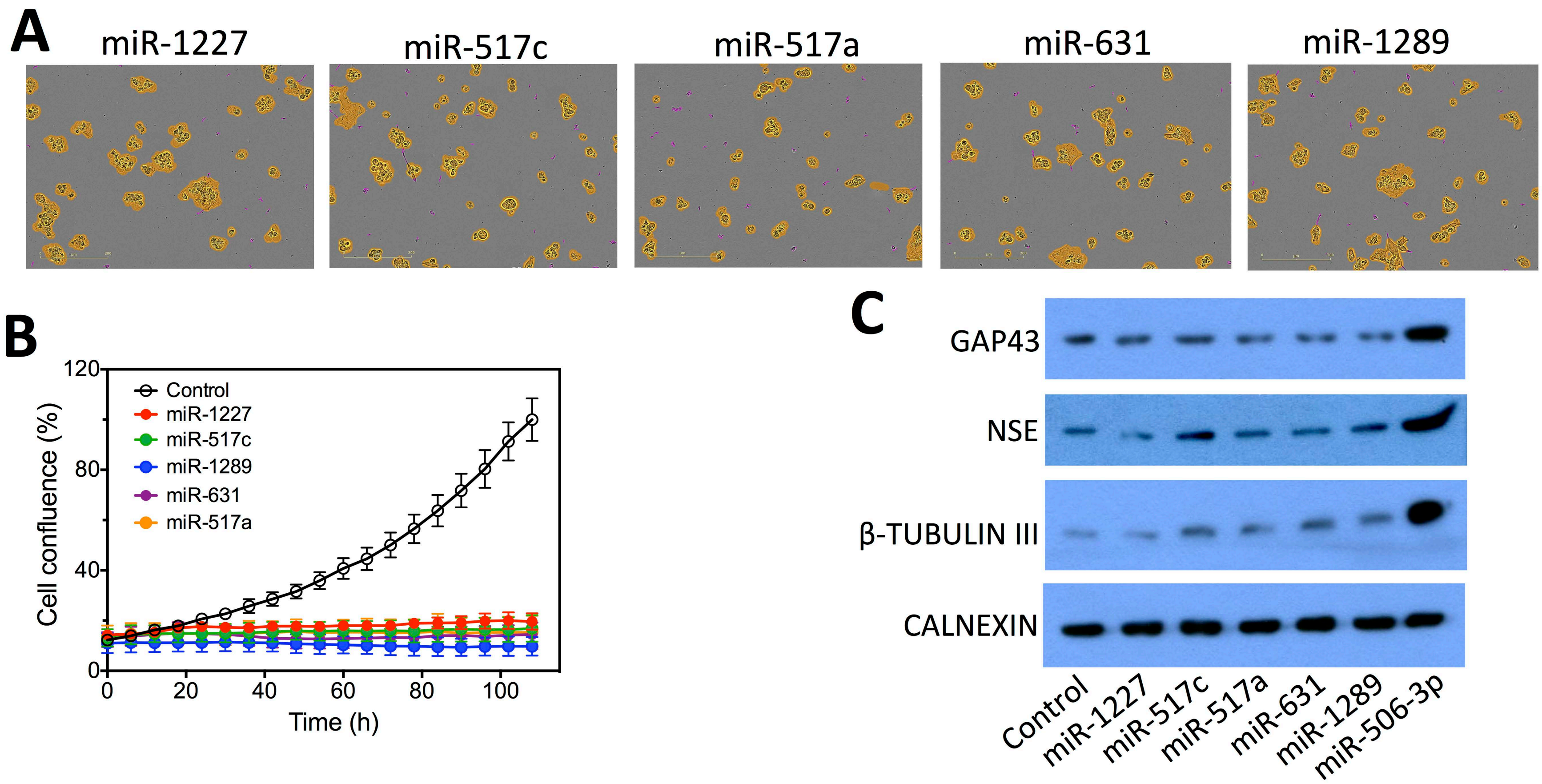

Suppl. Figure 6. miRNA mimics that induce BE(2)-C cell growth arrest but not differentiation were identified from HCS. A, 5 miRNA mimics that induce cell growth arrest but not neurite outgrowth. Shown are representative images analyzed to define neurites and cell body areas for the indicated miRNAs. **B,** Effects of the miRNA mimics on cell proliferation rate. BE(2)-C were transfected with 25nM of the indicated miRNA mimics or control oligo. Cell growth rates were analyzed as in Figure 2E. **C,** Effects of the miRNA mimics on expression of differentiation markers. BE(2)-C were transfected with 25nM of the indicated oligo. After 4 days, expression levels of the indicated proteins were examined by Western blots. miR-506-3p mimic was used as a positive control for inducing cell differentiation.

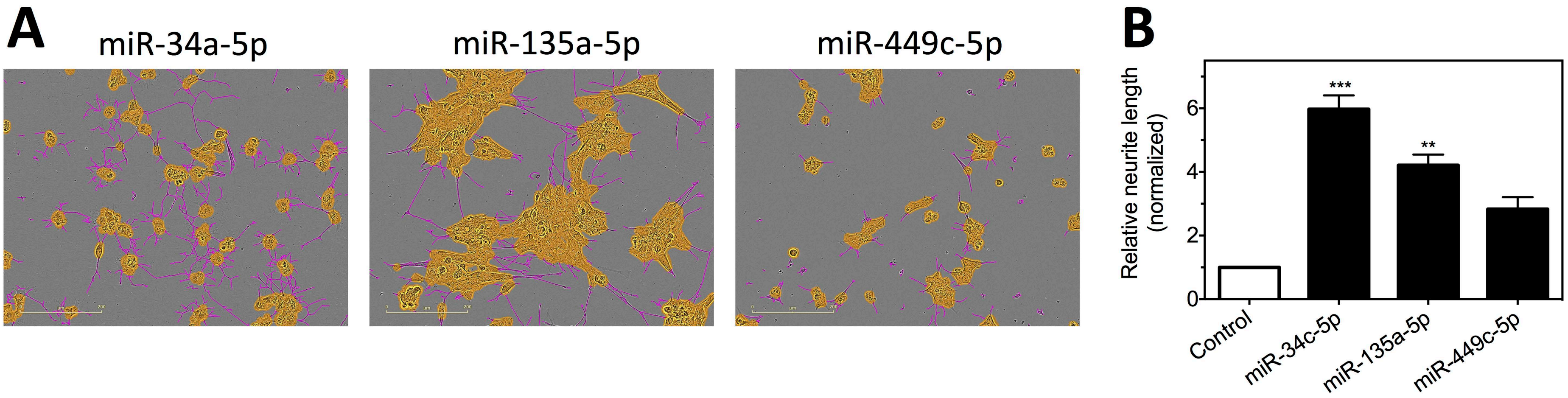

Suppl. Figure 7. Additional miRNA mimics identified as inducing neurite outgrowth from HCS. Shown are the representative images analyzed to define neurites and cell body areas (A), as well as quantification of neurite lengths (B). **, *p*<0.01; ****, *p*<0.0001.
